# Kappa-carrageenan and sodium alginate-based pH-responsive hydrogels for controlled release of methotrexate

**DOI:** 10.1098/rsos.231952

**Published:** 2024-04-24

**Authors:** Muhammad Anees Ur Rehman Qureshi, Nasima Arshad, Atta Rasool, Naveed Kausar Janjua, Muhammad Shoaib Butt, Muhammad Naqeeb Ur Rehman Qureshi, Hammad Ismail

**Affiliations:** ^1^ Department of Chemistry, Allama Iqbal Open University, Islamabad, Pakistan; ^2^ School of Chemistry, University of the Punjab, Lahore, Pakistan; ^3^ Department of Chemistry, Quaid-i-Azam University, Islamabad, Pakistan; ^4^ School of Chemical and Materials Engineering (SCME), National University of Science and Technology, Islamabad 44000, Pakistan; ^5^ Faculty of Sciences, Department of Zoology and Biology, Pir Mehr Ali Shah ARID Agriculture University, Rawalpindi, Pakistan; ^6^ Department of Biochemistry and Biotechnology, University of Gujrat, Gujrat, 50700 , Pakistan

**Keywords:** cancer, methotrexate, hydrogels, carrageenan, alginate, poly(vinyl alcohol)

## Abstract

Despite remarkable progress in medical sciences, modern man is still fighting the battle against cancer. In 2022, only in the USA, 640 000 deaths and 2 370 000 patients were reported because of cancer. Chemotherapy is the most widely used for cancer treatments. However, chemotherapeutics have severe physicochemical side effects. Therefore, we have prepared poly(amididoamine) dendrimeric carrageenan (CG), sodium alginate (SA) and poly(vinyl alcohol) (PVA) hydrogels by using solution casting methodology. The constituents of hydrogels were cross-linked by mutable quantity of 3-aminopropyl(diethoxy)methyl silane (APDMS). Hydrogels were characterized by Fourier transform infrared spectroscopy, thermal gravimetric analysis, scanning electron microscope and atomic force microscopy. Hydrogels exhibited higher swelling volumes in 5–7 pH range. *In vitro* biodegradation in ribonuclease-A solution and cytocompatibility analysis against DF-1 fibroblasts established their biodegradable and non-toxic nature, which enables them as a suitable carrier for chemotherapeutic compounds. Hence, methotrexate (MTX) as a model drug was loaded on CAP-8 hydrogel and its release was detected by the UV–visible spectrophotometer in phosphate-buffered saline (PBS) solution. In 13.5 h, 81.25% and 77.23% of MTX were released at pH 7.4 (blood pH) and 5.3 (tumour pH) in PBS, respectively. MTX was released by super case II mechanism and best fitted to zero-order and Korsmeyer–Peppas model. The synthesized APDMS cross-linked CG/SA/PVA dendrimeric hydrogels could be an efficient model platform for the effective delivery of MTX in cancer treatments.

## 1. Introduction

Although researchers have made tremendous progress in clinical research and pharmaceutical development, diseases like cancer are still almost incurable at their advanced stages. Cancer is the main cause of deaths worldwide. The cancer burden of the USA in 2022 was 2 370 000 while the death toll climbed up to 640 000 [1]. A comprehensive statistical investigation estimated about 20 million new cancer patients globally by 2025 [2]. It is a heterogeneous disease characterized by uncontrolled replication, invasion, proliferation, metastasis and angiogenesis in cancerous cells [3]. Genetic, reproductive, hormonal, nutritional and physical factors increase the risk of cancer [4,5]. The treatment and recovery of cancer patients is still a big challenge in present medical science. There are three common treatments for cancer, namely radiotherapy, surgery and chemotherapy [6]. Chemotherapy is the most widely used and effective method. However, smaller therapeutic indexes, drug resistance, adversarial drug reactions and reduced targeting greatly limited the widespread use of this treatment [7]. In addition, chemotherapeutic agents affect normal cells along with cancerous cells. Therefore, it is imperative to design carriers for therapeutic drugs which release drug at target site without affecting normal cells.

Hydrogel-based chemotherapeutic drug carriers are emerging for cancer treatments owing to their reduced side effects, and sustained and targeted delivery at tumour location, which greatly improves the bioavailability and efficacy of the drug [8]. In addition, hydrogels are biodegradable, non-toxic, soft-structured, porous, flexible and biocompatible in nature [9]. These are hydrophilic, three-dimensional polymeric frameworks that can retain a high amount of water without dissolving in it [10]. Swelling ability is a characteristic property of hydrogels that is affected by ionic strength, pH, osmotic pressure, heat, sound, and electric and magnetic fields [11].

Natural polysaccharides are the finest polymeric materials for hydrogel fabrication because of their bioavailability, composition, non-toxicity, elasticity and biodegradability [12]. Carrageenan (CG) is an anionic, linear, long chain and sulphated polysaccharide comprising 3,6-anhydro-d-galactose and d-galactose-4-sulphate repeating units [13]. It is extracted from red seaweeds. Owing to biodegradable, modifiable and biocompatible nature, CG-based hydrogels are eminent in biomedical and pharmaceutical industries [14]. On the basis of sulphate content, it is categorized into kappa, iota and lambda CG containing one, two and three sulphate groups per disaccharide, respectively [15]. Kappa-carrageenan (k-CG) is favoured for hydrogel fabrication because of the production of good quality gels with lesser sulphate content [16]. On the other hand, sodium alginate (SA) is obtained from brown seaweeds. It is a hydrophilic, water-soluble, degradable, biocompatible, abundant and low-cost polymer that absorbs water readily [17]. It has demonstrated excellent compatibility with CG, so SA is widely used in hydrogel formulations. SA-incorporated hydrogels have improved and higher porosity levels [18].

Synthetic polymers are chosen in the biomedical sector because of ease in processing, degradability and superior mechanical performances [19]. Therefore, structural modifications of biopolymers are carried out by cross-linking them with synthetic polymers. Poly(vinyl alcohol) (PVA) is a whitish, water-soluble, odourless synthetic polymer used as stabilizer, thickener, additive and coating material [20]. In addition, PVA yields cytocompatible hydrogels because of low adhesive property for proteins [21]. Consequently, it is used in stents, ocular drops, cartilage replacements, ophthalmic lubricants, etc. [22]. Incorporation of PVA improves the mechanical resilience and physicochemical properties of hydrogels.

Methotrexate (MTX) is a chemotherapeutic drug used for the treatment of breast, brain, lung and blood cancer [23]. Further, it is antirheumatic and anti-inflammatory drug administered for rheumatoid arthritis, psoriasis and auto-immune diseases [24–26]. MTX has poor water solubility and severe side effects like nausea, skin rashes, tiredness, lung infection, low white cell count, kidney disorders, etc. [27]. Furthermore, only 42%–57% of MTX binds to the serum [28]. Therefore, highly branched and multifunctional polyamidoamine (PAA) dendrimer was also incorporated in hydrogels, which assists in drug solubility, bioavailability and controlled release.

Herein, biopolymer (CG and SA) and synthetic polymer (PVA) are blended followed by the addition of PAA dendrimer (generation 0.0). Cross-linking among hydrogel components is achieved by different quantities of 3-aminopropyl(diethoxy)methylsilane (APDMS). Hydrogels were characterized and their swelling behaviours, cell viabilities and *in vitro* biodegradation test were also carried out. MTX-loaded hydrogels were subjected to *in vitro* release at pH 5.3 and 7.4 in phosphate-buffered saline (PBS) solution, which represented sustained MTX release.

## 2. Experimental

### 2.1. Chemical and solvents

k-CG (MW: 672 000 g mol^−1^, CAS No. 11114-20-8, 15.3% sulphate content), APDMS (97%, MW: 191.34 g mol^−1^, CAS No. 3179-76-8), PAA (MW: 516.68, generation 0.0, *d* = 0.854 g ml^−1^l, CAS No. 155773-72-1), MTX (MW: 454.44 g mol^−1^, CAS No. 59-05-2) and ribonuclease-A from bovine pancreas (MW: 13 700 g mol^−1^, powder, CAS No. 9001-99-4) were procured from Sigma Aldrich (Darmstadt, Germany). SA (MW: 60 275 g mol^−1^, food grade, purity 99.99%) was imported from Dabur, India Limited. PVA (MW: 65 000 g mol^−1^, CAS No. 9002-89-5, polymerization degree 1500) was acquired from Duskan. In addition, methanol (MW: 32.04 g mol^−1^, *d* = 0.79 g ml^−1^), ethanol (MW: 46.07 g mol^−1^, HPLC grade, *d* = 0.79 g ml^−1^) and *N,N*-dimethylformamide (MW: 73.10 g mol^−1^, 99.8%, *d* = 0.95 g ml^−1^) were obtained from Scharlau, Spain. Sodium chloride (MW: 58.44 g mol^−1^), sodium azide (MW: 65.01 g mol^−1^), sodium hydroxide (MW: 40 g mol^−1^), calcium chloride (anhydrous, MW: 110.98 g mol^−1^) and potassium chloride (MW: 74.55 g mol^−1^) were purchased from Daejung, Korea.

### 2.2. Hydrogel synthesis

APDMS cross-linked hydrogels were prepared by blending natural (k-CG and SA) and synthetic (PVA) polymers by solution casting technique. k-CG (0.25 g) and SA (0.25 g) were separately dissolved in 30 ml of distilled water. Both solutions were mixed and stirred for 1 h. Likewise, PVA (0.5 g) was dissolved in 30 ml of distilled water and transferred into afore-mentioned polymeric solution and blended for 1.5 h. In the next step, PAA (60 µl) was diluted in 3 ml of methanol and poured into the above blending mixture followed by stirring for more than 1 h. Subsequently, different amounts of APDMS (80, 160, 240 and 320 µl) were diluted in 5 ml of ethanol and added to the blending mixture. Then, after agitation up to 3 h at 70°C the mixture was poured over Petri plates and dried at room temperature. Hydrogels were named as CAP (control), CAP-8, CAP-16, CAP-24 and CAP-32. CAP (control) does not contain APDMS, while CAP-8, CAP-16, CAP-24 and CAP-32 comprise 80, 160, 240 and 320 µl of APDMS cross-linker, respectively. The entire process of hydrogel fabrication was accomplished at 70°C under incessant stirring.

### 2.3. Hydrogels characterization

The dried hydrogels were examined by Nicolet iS10 Fourier transform infrared (FTIR) instrument built by Thermo Fisher Scientific (Waltham, MA, USA). The FTIR spectra of hydrogels, pure drug and drug-loaded hydrogel were recorded from 400 to 4000 cm^−1^. Thermal analysis of hydrogels was performed by using thermogravimetric analysis (TGA) model Q50 marketed by Thermal Analysis (TA) instrument (New Castle, DE, USA). The instrument was fitted with a platinum pan in which 5 mg of hydrogel specimen was heated from 25 to 700°C at a heating rate of 10°C min^−1^ under an inert atmosphere. The inert atmosphere was attained by non-stop nitrogen purging at 40 ml min^−1^ flow rate. For the morphological study by scanning electron microscopy (SEM), each hydrogel specimen was coated with tungsten metal by using a sputter coater made by Safematic model CCU-010. The coating was performed up to 1.5 min while the coating rate was 0.8 nm s^−1^. Then, surface examination of hydrogels (upper shiny surface) was performed at different magnifications with the help of SEM model MIRA3 produced by TESCAN (Brno, Czech Republic). The atomic force microscopy (AFM) was carried out to obtain high-resolution surface imaging of hydrogels using a mechanical probe. The non-tap scanning mode by means of the JEOL scanning probe microscope (Japan model JSPM-5200) coupled with a cantilever made up of silicone doped with chromium and cobalt metals were used for AFM analysis.

### 2.4. Cell viability assay

DF-1 fibroblast cells were grown in Dulbecco's modified eagle medium (DMEM) and 10% fetal bovine serum (FBS) was added as nutrient media in them. The cell viability of hydrogels was determined by MTT assay against DF-1 fibroblast cell lines. The DF-1 fibroblast cells (1 × 10^4^) were seeded over sterile hydrogels (4 mg) followed by their incubation at 37°C. DF-1 cells devoid of hydrogel were used as standard reference. After 24 and 48 h, absorbance values were detected by means of a Bio-Rad built microplate reader model PR4100 at 630 nm. The experimental outcomes are represented in average of three experiments using equation (2.2).


(2.1) $$cell\;viability\;(\%)=\frac{sample\;absorbance}{reference\;absorbance}\times 100.$$

### 2.5. *In vitro* biodegradation

First of all, 0.25 mg ml^−1^ of ribonuclease-A enzyme solution was formed in PBS solution. Calcium chloride (0.3 g) and sodium azide (0.02 g) were added to the above PBS solution to inhibit microbial growth. Then, a predetermined amount (50 mg) of each hydrogel was placed in 30 ml prepared ribonuclease-A enzyme solution. The hydrogels were taken out from the solution after 1st, 3rd and 7th day. Their surface water was removed with the aid of filter paper. Subsequently, the mass loss of each hydrogel was computed by the subtraction of final weight from initial weight [29]. The *W*_*i*_ is the initial mass of dried hydrogel (50 mg) while *W*_*f*_ is the mass of hydrogel taken from ribonuclease-A solution after 1st, 3rd and 7th day.


(2.2) $$Biodegradation\;\left(\%\right)=\frac{W_{i}-W_{f}}{W_{i}}\times 100.$$

### 2.6. Swelling ability

Swelling abilities of hydrogels were studied in distilled water, buffer, non-buffer and electrolytic (NaCl and CaCl_2_) solutions. For swelling analysis, 10 mg of each hydrogel was engrossed in distilled water and the above-mentioned aqueous solutions. After regular time pauses, hydrogels were removed from the solution. The water on their surface was removed by tissue paper followed by measurement of swollen weight [30]. Swelling ability was calculated from equation (2.3) shown below.


(2.3) $$Swelling\;ability\;\left(\%\right)=\frac{W_{s}-W_{d}}{W_{d}}\times 100.$$

*W*_*s*_ and *W*_*d*_ represent swelled and dried mass of hydrogel samples, correspondingly. All the swelling experimentations were conducted at room temperature in triplicates. The deviances in the results were computed and reflected as error bars in graphical outcomes.

### 2.7. Methotrexate loading in hydrogel

MTX was loaded on CAP-8 hydrogel in five steps. k-CG (0.25 g), SA (0.25 g) and PVA (0.5 g) were separately dissolved in 30 ml of distilled water. In the first step, k-CG and SA solutions were mixed and agitated for 1 h. In the second step, PVA solution was poured into the polymeric solutions. The blending mixture was stirred for an additional 1.5 h. In the third step, PAA (60 µl) was dispersed in methanol (3 ml) and decanted into the above polymeric mixture. Afterwards, the mixture was agitated for 1 h. In the fourth step, MTX (50 mg) was taken in 10 ml of *N,N*-dimethylformamide and transferred to the afore-mentioned mixture. In the fifth step, APDMS (80 µl) was diluted in ethanol (5 ml) and added to the blend. Afterwards, the content was stirred for 3 h, cast over Petri plates and dried at 70°C. The MTX-loaded CAP-8 was coded as MCAP-8.

### 2.8. Encapsulation efficiency

The efficiency of MTX loading on hydrogel was also determined by the placement of MTX-loaded hydrogels in PBS (50 ml) at pH 7.4. After 24 h, solutions were agitated for 20 min and subjected to UV–visible spectrophotometer at 302 nm. From a standard calibration, curve quantity of MTX loaded in hydrogel was computed.


(2.4) $$EE\left(\%\right)=\frac{Q_{MTX}}{Q_{total}}\times 100,$$

where EE is the encapsulation efficiency; *Q*_MTX_ is the actual quantity of MTX encapsulated, and *Q*_total_ is the theoretical amount of MTX.

### 2.9. Methotrexate release and kinetics

MTX-loaded CAP-8 (MCAP-8) release studies were carried out in freshly prepared and sterilized 1 M PBS at pH 7.4 (blood pH) and 5.3 (tumour pH). MCAP-8 was added in 200 ml of PBS and absorbance values were determined every 30 min at 302 nm by using an 1800 UV–visible spectrophotometer manufactured by Shimadzu (Kyoto, Japan). Pure PBS was used as reference and MTX released was computed by the calibration curve. The drug release kinetic studies were also studied by using the following six models:

1. zero-order model


(2.5) $$M_{t}=M_{o}+K_{o}t$$

2. first-order model


(2.6) $$logM_{t}=logM_{o}-\frac{kt}{2.303}$$

3. Korsmeyer–Peppas model


(2.7) $$ln\frac{M_{t}}{M_{o}}=nlnt+lnk$$

4. Higuchi model


(2.8) $$ft=Q=K_{H}\times t^{1/2}$$

5. Hixon–Crowel model


(2.9) $$ft=Q=K_{H}\times t^{1/2}$$

6. Baker–Lonsdale model


(2.10) $$F_{t}=\frac{3}{2}[1-\begin{array}{l} {\left(1-\frac{M_{t}}{M_{o}}\right)}^{\frac{2}{3}}]\frac{M_{t}}{M_{o}}=K(t{)}^{0.5} \end{array}$$

where *M*_o_ is the total drug quantity and *M*_*t*_ is the drug released at time *t*. In addition, *K*_*H*_, *K* and *K*_o_ are rate constants.

### 2.10. Statistical analysis

Origin Pro (8.5.1) developed by Origin Lab Corporation (Northampton, MA, USA) was used for statistical examination of the data. In addition, linear fitting was used to evaluate swelling as well as kinetic parameters for MTX release from hydrogels. The deviances in statistical data were carried out by one-way ANOVA. *p* < 0.05 was considered significant.

## 3. Results and discussion

### 3.1. Scheme

**Figure 1.**
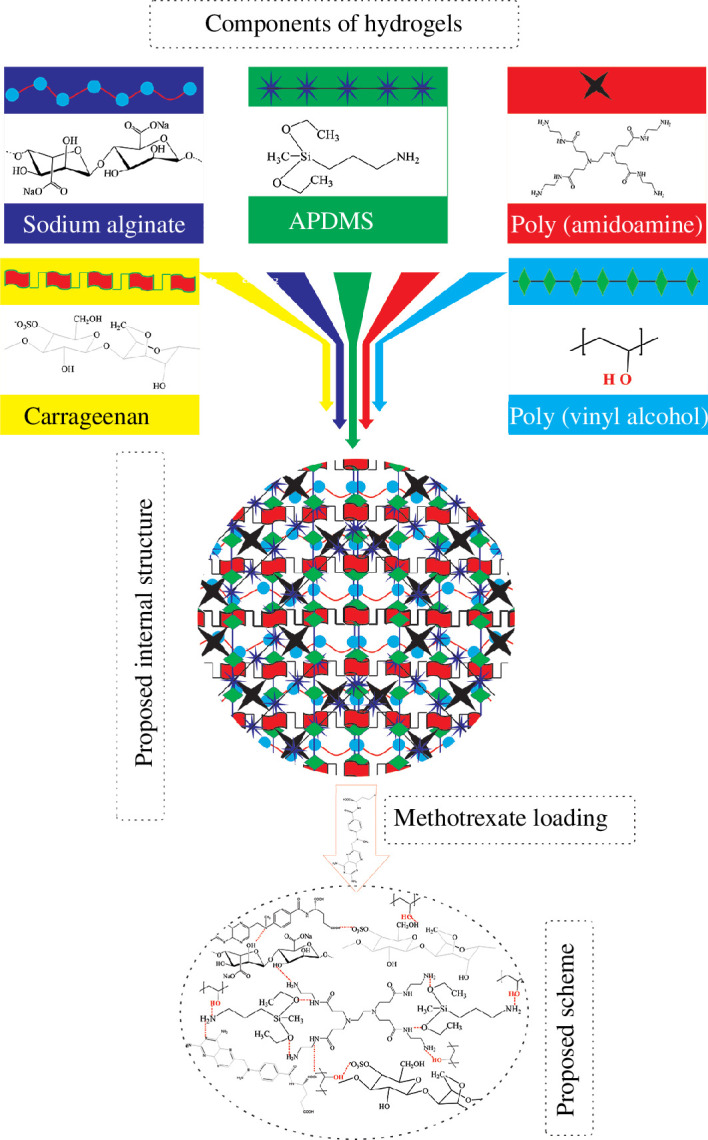
The proposed structural scheme for APDMS cross-linked CG/SA/PVA/PAA hydrogels for MTX release.

In figure 1, the proposed scheme and internal possible structure are provided for APDMS cross-linked CG/SA/PVA/PAA hydrogels. Intermolecular forces, mainly hydrogen bonding, are accountable for the development of hydrogel architecture. Hydrogen bonding exists among hydroxyl and carboxyl groups that are present in CG, SA and PVA. In addition, APDMS cross-linker also takes part in intermolecular interaction via amino (–NH_2_) and ethoxy (–OC_2_H_5_) functionalities. Amino groups interact with sulphate and hydroxyl and carboxyl functionalities to produce hydrogels as shown in figure 1.

### 3.2. Fourier transform infrared analysis

**Figure 2.**
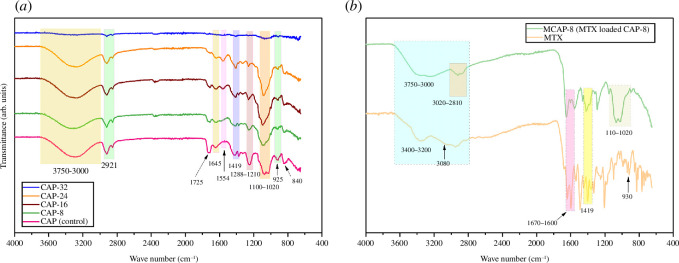
FTIR spectrum (*a*) formed hydrogels (*b*) MCAP-8 and pure MTX.

Fourier transform infrared (FTIR) analysis was used to confirm the characteristics functional groups of each constituent used for hydrogel synthesis. In addition, developments of interactions for the generation of hydrogel interfaces are also confirmed by the FTIR technique. The fabricated hydrogels comprised CG, SA, PVA, PAA and APDMS, and their FTIR spectra are shown in figure 2*a*.

The presence of CG is confirmed by the –OH stretching, sulphate ester, 3,6-anhydro-d-galactose and d-galactose-4-sulphate from the peaks at 3000–3400, 1210–1288, 925 and 840 cm^−1^, respectively [31]. SA contains hydroxyl and carboxyl functionalities. The peaks for the hydroxyl group were observed at 3400 cm^−1^ while carbonyl (C=O) and COO symmetric stretches appeared at 1725 and 1419 cm^−1^, correspondingly [32]. The presence of PVA is confirmed by the broad –OH stretching from 3000 to 3700 cm^−1^. Similarly, the bands at 2921 and 1554 cm^−1^ certified the C–H stretching and amino groups in PAA, respectively [33]. Fabrication of the hydrogels was ascertained from siloxane linkages (Si–O-Si and Si–O–C) in the region of 1020–1100 cm^−1^ owing to APDMS cross-linking.

FTIR spectrum of MTX and MCAP-8 are reflected in figure 2*b*, where MTX is characterized by the characteristic absorption peaks related to the hydroxyl, primary amine and carboxyl group. The peak at 3450 cm^−1^ corresponded to the hydroxyl stretching; however, N–H stretching owing to the primary amine was also noted at 3080 cm^−1^, which also overlapped in the region of –OH stretching. The region from 1600 to 1670 cm^−1^ and 1500 to 1550 cm^−1^ belongs to the carbonyl stretching N–H bending vibrations, correspondingly [34]. The peak at 1419 cm^−1^ is because of the symmetric stretching of COO^−^ while hydroxyl bending is noticed at 930 cm^−1^. Hence, FTIR confirmed the MTX loading in CAP-8.

### 3.3. Thermal gravimetric analysis

**Figure 3.**
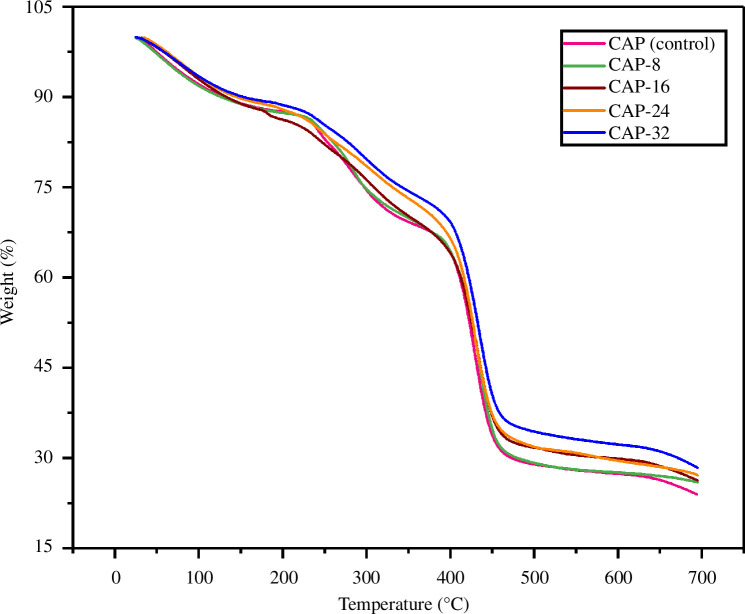
Thermogram of prepared hydrogels.

The thermal gravimetric analyses of hydrogels were performed to confirm the development of binding forces imparted by the APDMS in hydrogels. The results are displayed in figure 3. TGA curves reflected three-phase decomposition in each hydrogel framework. The first deterioration starts from 50 to 200°C and is ascribed by the elimination of moisture and bound water in hydrogels. The second phase of decomposition ranging from 200 to 400°C is explained by the condensation of hydroxyl moieties in CG, PVA and SA skeleton. Furthermore, CG de-sulphation has occurred in this zone as well [35]. The decomposition in the PVA structure is another reason for the mass loss in this zone [36]. The third and final region ranging from 400 to 700°C is accredited to the breakdown in the backbone of the polymer [37]. It was observed that an increase in APDMS quantity improved the thermal stability of hydrogels, which is also evident from recent studies [38,39].

APDMS is a silane cross-linker that possesses three cross-linking sites. One amino and two ethoxy groups are present per molecule, which interacts with the functional groups of polymers. Therefore, flexible and non-rigid cross-linking takes place among the components of hydrogels, namely CG, SA, PVA and PAA dendrimer. Therefore, cross-linking is proportional to the APDMS amount. CAP (control) lacks APDMS, whereas CAP-8, CAP-16, CAP-24 and CAP-32 contain 80, 160, 240 and 320 µl APDMS, respectively. This is the cause of maximum thermal resilience in CAP-32. The numerical data of weight loss at different temperatures are given in table 1. It is also inferred that thermal, biodegradation and drug release of hydrogels can be tuned by APDMS cross-linking.

**Table 1.** The weight loss (%) of hydrogels as a function of temperature.

hydrogels	100°C	200°C	300°C	400°C	500°C	600°C
CAP (control)	92.13	87.51	74.56	64.17	28.95	27.37
CAP-8	91.84	87.36	74.73	64.41	29.19	27.57
CAP-16	92.97	86.24	76.27	64.08	31.73	29.85
CAP-24	93.44	87.91	78.56	66.48	31.86	29.54
CAP-32	93.45	88.67	79.69	69.22	34.40	32.23

### 3.4. Scanning electron microscopy analysis

**Figure 4.**
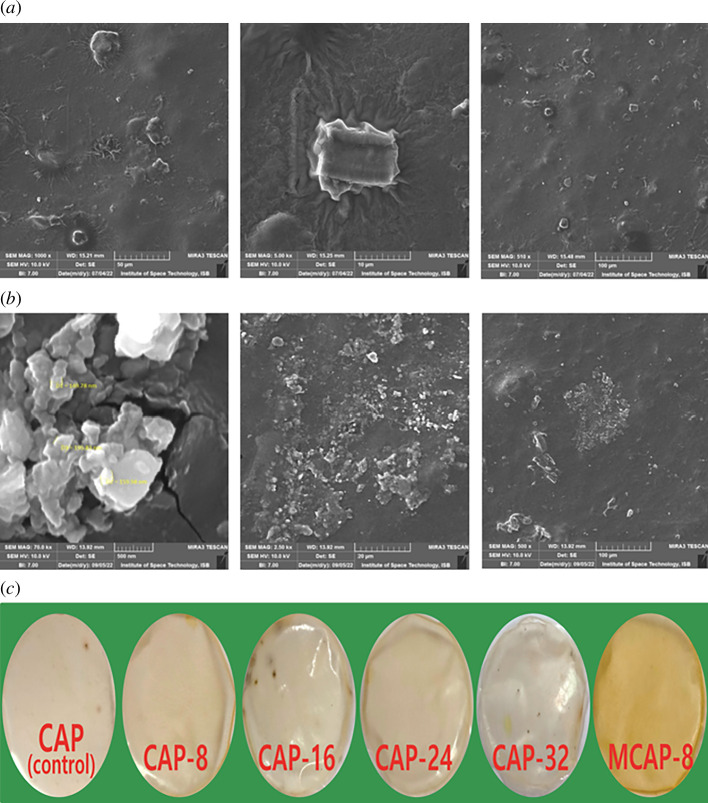
(*a*). SEM micrograph of CAP-8 at different magnifications. (*b*). SEM images of MCAP-8 (MTX-loaded CAP-8). (*c*). The pictures of fabricated CG-based hydrogels.

In figure 4*a*, the microstructure of CAP-8 hydrogel was observed by field emission MIRA3 TESCAN that demonstrated dense, rough, complex, compact and net-like morphology, which enhances surface area and aids in drug adsorption/release. These microstructures and small-sized multifaceted organizational sites are preferred for drug loading/release because their solvent and drug retentions for longer period thus present sustained release [40].

On other hand figure 4*b* presents SEM micrograph of MCAP-8 that describes the presence of MTX molecules on the surface of CAP-8. In addition, the surface of MCAP-8 is more heterogeneous, porous and multifaceted. The SEM analyses not only confirmed the effective blending and synthesis of hydrogels but also confirmed the MTX loading on CAP-8. The SEM micrographs endorsed the compatibility of hydrogel components for drug delivery applications. The pictures of CAP (control), CAP-8, CAP-16, CAP-24, CAP-32 and MCAP-8 are shown in figure 4*c*, which presents the flat and amorphous nature of fabricated hydrogels.

### 3.5. Atomic force microscopy analysis

**Figure 5.**
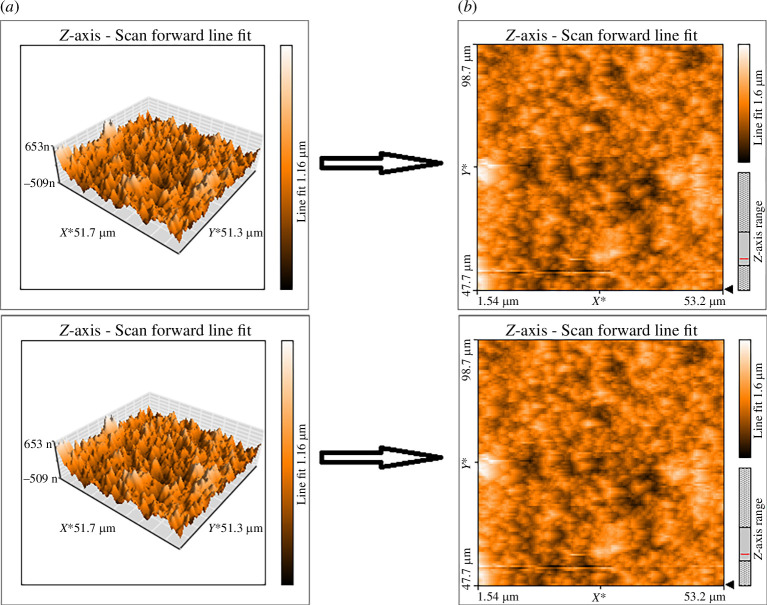
Topographic analysis by AFM (*a*) CAP-8 and (*b*) MCAP-8.

The micrographs of CAP-8 and MCAP-8 obtained from the scanning probe microscope are given in figure 5. The micrograph in figure 5*a* confirmed the fabrication of amorphous, porous and smooth surface of CAP-8 specimen. In figure 5*b*, MCAP-8 demonstrated heterogeneous, irregular, humpy and rough surfaces owing to the adsorbed MTX on its surface. These uneven surfaces are the sites that provide high surface area and are the sites for solvent absorption inside the hydrogel matrix. Consequently, the loaded drug molecules are released [41].

### 3.6. Cell cytocompatibility test

**Figure 6.**
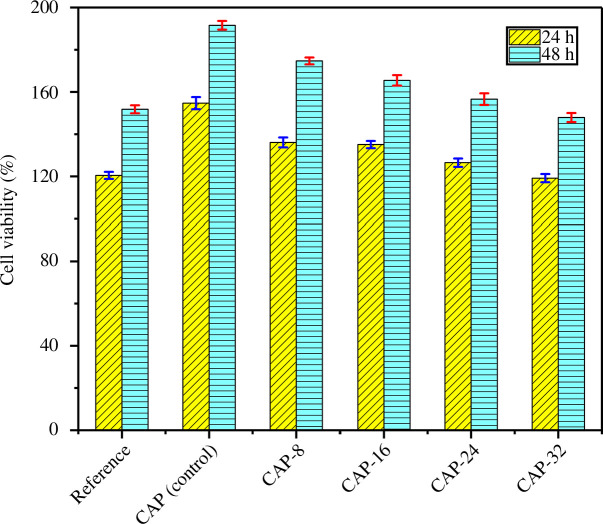
The viability of the fabricated hydrogels against DF-1 fibroblast cells.

The primary requirement of hydrogel-based carrier is their biocompatibility and non-toxicity for living cells. Henceforth, the MTT assay was performed against DF-1 fibroblast cell lines, and the results are given in figure 6. It was revealed that CAP (control), CAP-8, CAP-16, CAP-24 and CAP-32 demonstrated higher cell viabilities as compared with the reference (DF-1 fibroblasts without hydrogel). In addition, a favourable atmosphere was provided by the hydrogels that resulted in a significant multiplication of cells. This behaviour suggests the suitability and non-toxicity of hydrogels for drug delivery, tissue engineering and other biomedical applications. By the increase in the incubation period, cell viabilities were also enhanced. In the same way, an increase in APDMS quantity has an inverse effect in the growth of fibroblast cells. This behaviour is explained by the cross-linking capability of APDMS which is proportional to its amount. Thus, stronger binding imparted by APDMS in hydrogels reduces the biocompatibility of hydrogel samples for cell growth.

### 3.7. *In vitro* biodegradation studies

Biodegradable hydrogels are preferred in medico-biological applications because of no requirements for their removal after the release of encapsulated therapeutic drugs [42]. In addition, tunable degradation in hydrogels is used to control drug release as another mode of drug release from the hydrogel framework [43]. Biodegradation capabilities of the prepared hydrogels are attributed to the existence of biodegradable polymers, that is, CG, SA and PVA.

**Figure 7.**
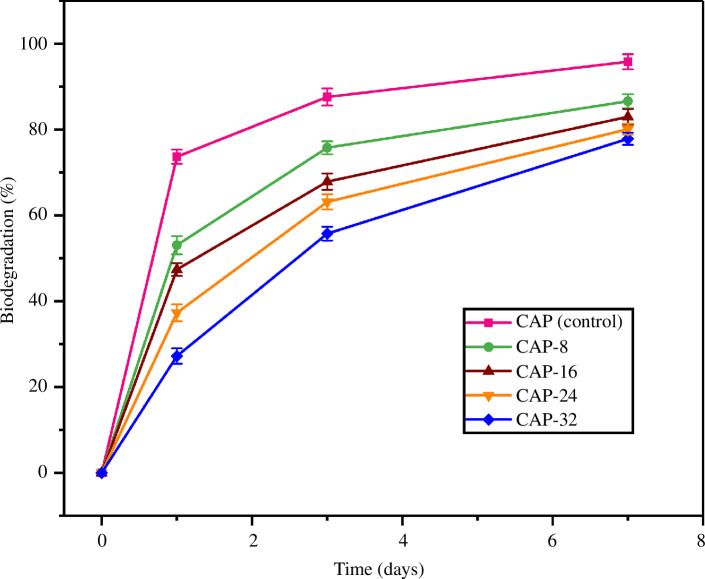
Biodegradation behaviour of the hydrogels in ribonuclease-A solution.

PVA and SA have shown excellent biodegradability [44,45]. On the other hand, CG is composed of *β*-1,4 and *α*-1,3-glycosidic linkages among monomers as depicted in FTIR. The intermolecular forces, hydrogen bonding and siloxane interactions confirmed by the FTIR could be tainted by the *in vivo* enzyme actions [46]. Therefore, biodegradational rates of hydrogels were carried out in ribonuclease-A solution which was prepared in PBS. As a result, CAP (control), CAP-8, CAP-16, CAP-24 and CAP-32 revealed 95.8%, 86.6%, 82.9%, 80% and 77.8% degradation in 7 days as shown in figure 7. These results not only confirmed that synthesized hydrogels are degradable but also endorsed their use for drug release and biomedical applications. Moreover, the degradation of hydrogels decreased by the increase in the amount of APDMS as it has three cross-linking points which are accountable for the formation of strong hydrogen and covalent bonding in the hydrogel.

### 3.8. Swelling in distilled water

**Figure 8.**
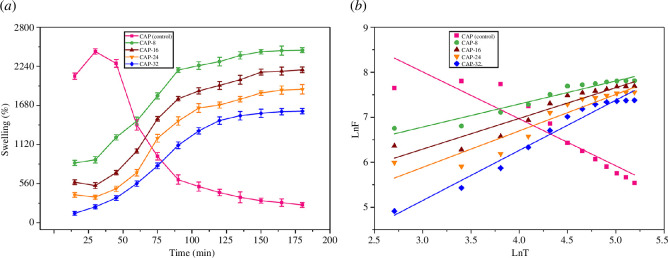
(*a*) Swelling behaviour of hydrogels in distilled water (*b*) calibration for computing diffusion parameters.

Hydrogels captivate water because of their hydrophilic nature. Therefore, the swelling capabilities of hydrogels were studied in distilled water, and the results are given in figure 8*a*.

It is observed that all hydrogel specimens reflected a linear increase in swelling with respect to time except CAP (control) which initially depicted higher swelling percentage up to 30 min as compared with other hydrogels. CAP (control) does not contain APDMS cross-linker; as a result, after 30 min its swelling abilities were significantly reduced.

On the other hand, CAP-8, CAP-16, CAP-24 and CAP-32 have shown time-dependent swelling and the constituents of hydrogels remained intact owing to the APDMS cross-linking. Among APDMS cross-linked hydrogels, CAP-8 exhibited the highest swelling (2471%) while the least swelling percentage was shown by CAP-32 (1598%). Thus, APDMS inversely affected the swelling aptitude of hydrogels because it contains three cross-linking points that participate in stronger hydrogen and covalent bond formation which not only decreased the swelling percentage but also improved stabilities in water.

The unique swelling action supports the exploration of prepared hydrogels as carrier in drug delivery. The entry of water inside hydrogels is governed by the process of diffusion, which is explained by equation (3.1), where, *F* is the fractional swelling; *k*, swelling rate constant; *t,* the time taken to swell; and *n*, the swelling exponent. The values of *k* and *n* were calculated from standard calibration curves and their numerical values are given in table 2. The standard curves for each hydrogel formulation are shown in figure 8*b*.

**Table 2.** Diffusion factors are calculated from the standard calibration curves.

parameter	CAP (control)	CAP-8	CAP-16	CAP-24	CAP-32
Adj. *R*-square	0.84775	0.93498	0.89438	0.88438	0.96749
*R*-square (COD)	0.86159	0.94089	0.90399	0.89489	0.97045
Pearson’s *r*	−0.92822	0.97	0.95078	0.94599	0.98511
residual square’s sum	1.09996	0.10334	0.31088	0.48494	0.23786
slope (*n*)	−1.04383	0.51165	0.68247	0.81055	0.98511
s.e.	0.1323	0.04055	0.07033	0.08785	0.06152
intercept	11.14022	5.24697	4.24387	3.45421	1.80479
*k*	68886.8	189.99	69.677	31.6333	6.07869

s.e. is the standard error in the slope.

COD is the coefficient of determination


(3.1) $$F=kt^{n}$$

It is clear from the data that solvent molecules diffused into hydrogel matrix. As the value of *n* for hydrogels is in accordance with 0.5 < *n* < 1 that indicated anomalous non-Fickian diffusion was followed by water molecules [47].

### 3.9. Swelling in non-buffers and buffers

**Figure 9.**
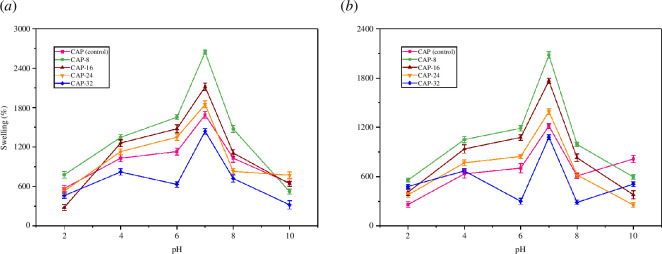
Swelling response of synthesized hydrogels from pH 2 to 10 (***a***) buffers (***b***) non-buffers.

The swelling of hydrogels is highly influenced by the pH of the medium. In addition, different parts of the body also have different pH. The pH-targeted release is explored in the delivery of different classes of anti-bacterial, anti-cancerous and anti-inflammatory drugs. Thus, the extent of hydrogel swelling was determined in non-buffer and buffer solutions at pH 2, 4, 6, 7, 8 and 10. Non-buffer solutions were made by aqueous solutions of 0.1 M NaOH and 0.1 M HCl. The buffer solutions were prepared by using KCl, KH_2_PO_4_, NaH_2_PO_4_, Na_2_HPO_4_, CH_3_COOH, CH_3_COONa, NaOH and HCl. The pH was adjusted by the pH meter model HANNA instrument-8417. The results confirmed pH-sensitive swelling of the prepared hydrogels in non-buffer and buffer solutions, represented in figure 9*a,b* respectively.

CAP (control), CAP-8, CAP-16, CAP-24 and CAP-32 hydrogels exhibited lesser swelling at highly acidic and basic pH. Moreover, the hydrogels' swelling percentages were high from pH 5, and maximum swelling was presented at pH 7. This unique behaviour of hydrogels suggests their potential as carrier for anti-cancerous drugs. CG is an anionic polymer comprised of hydroxyl and sulphate functional groups. On the other hand, hydroxyl groups in SA, PVA and amino groups found in PAA dendrimer and APDMS are also protonated at low pH. As a result, stronger electrostatic interactions are generated inside hydrogel frameworks among anionic sulphate and cationic (protonated) amino group (–SO_4_^-^……NH_3_^+^) that inhibited the flow of solvent molecules into the hydrogel matrix [48].

At neutral pH, the amino groups of PAA and APDMS are deprotonated, which decreased the strength of intermolecular force; for that reason, the water captivation of hydrogels was increased. When pH increases from 7, the complexation and charge imbalance are produced, which probably compacts hydrogel and decreases swelling volumes. In comparison with non-buffers, the swelling action of hydrogels is comparatively lower in buffers even at the same pH; however, swelling pattern is the same. For instance, CAP-8 depicted 2645% and 2080% swelling in non-buffer and buffer solutions, correspondingly. The higher ionic strength in the buffer medium is the cause of the relative decrease in swelling.

### 3.10. Swelling in ionic solutions

The presence of ions and charges has a direct impact on the hydrogel’s swelling capabilities.

**Figure 10.**
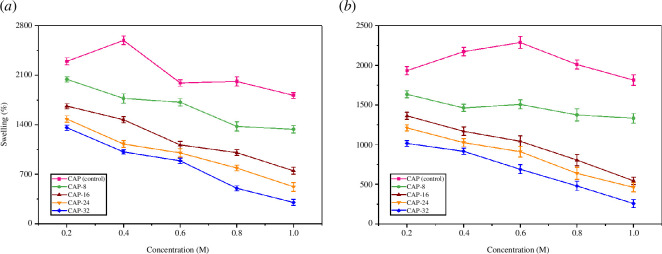
Swelling percentage of the hydrogels (*a*) NaCl (*b*) CaCl_2_.

Swelling percentage of CAP (control), CAP-8, CAP-16, CAP-24 and CAP-32 at variable molar concentration of NaCl and CaCl_2_ are depicted in figure 10*a,b*, accordingly. The decrease in swelling % is observed by the upsurge in the number of ions owing to the rise of osmotic pressure within the hydrogel complex. When osmotic pressure inside hydrogel is raised, it restricts water absorption [49]. In NaCl, higher swelling volumes are detected relative to CaCl_2_. For example, in NaCl and CaCl_2_ solution of 0.4 M, CAP-8 demonstrated 1771% and 1464% swelling. This is explained by the variations in ionic charge. In contrast to Na^+^, Ca^2+^ is a bivalent cation which causes stronger interchain complexation with amino group of PAA dendrimer. As a result, lesser swelling percentage was observed in CaCl_2_ as compared with NaCl. In addition, greater ionic charge generates compactness in hydrogel structure because of interchain linkages [50].

### 3.11. *In vitro* encapsulation efficiency of hydrogels

**Figure 11.**
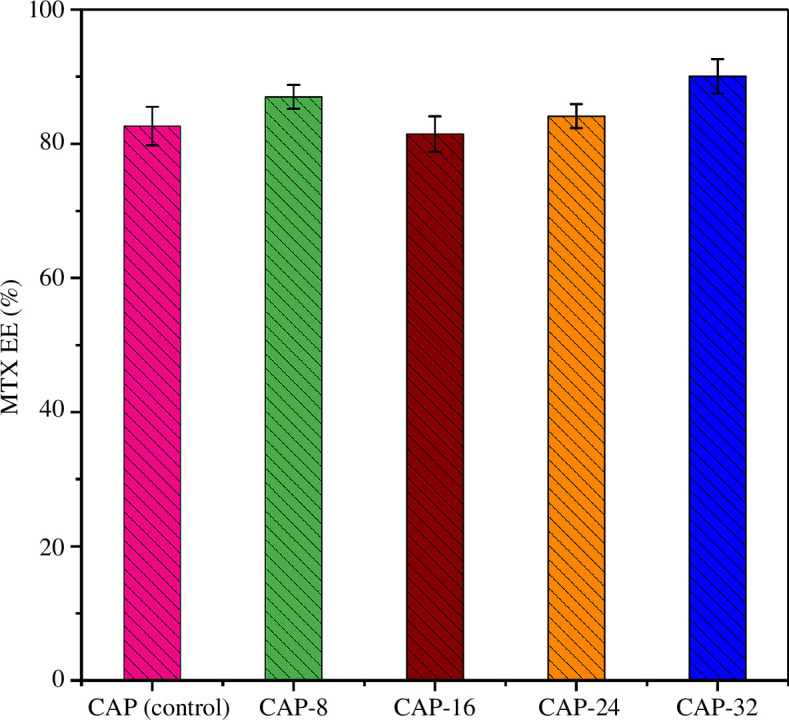
EE% of APDMS bound CG/SA/PVA/PAA hydrogels.

MTX loading in the hydrogel specimen was also elucidated and the results of MTX encapsulation are displayed in figure 11. All hydrogel formulations depicted good encapsulation capabilities for MTX. As encapsulation efficiency (EE) % of CAP (control), CAP-8, CAP-16, CAP-24 and CAP-32 are 82.65% ± 2.86, 87% ± 1.77, 81.46% ± 2.66, 84.12% ± 1.8 and 90.06% ± 2.57, respectively.

### 3.12. *In vitro* methotrexate release

**Figure 12.**
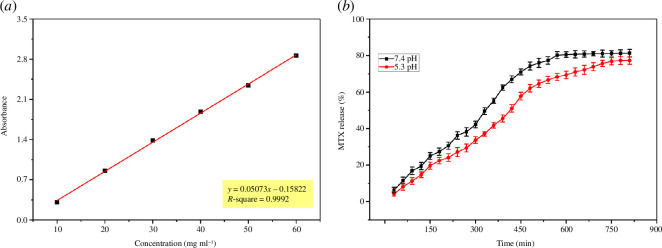
(*a*) Standard calibration curve for MTX (*b*) MTX release pattern.

MTX (50 mg) was loaded on CAP-8 hydrogels (coded as MCAP-8) because of good swelling and cytocompatibility. PBS solution was prepared and sterilized by autoclave. The pH of PBS was adjusted to 7.4 and 5.3. The MTX release was investigated at the pH of blood (7.4) and tumour tissues (5.3). In the subsequent step, MCAP-8 was placed in 200 ml PBS and the absorbance values were recorded at 302 nm after every 30 min using Shimadzu UV–visible spectrophotometer model 1800 made in Japan. PBS solution was used as a reference. The concentration of MTX release was computed by the standard curve shown in figure 12*a*.

Figure 12*b* depicts that initially MTX release from MCAP-8 is primarily controlled by the swelling, so a faster release of MTX was detected. In the first 4 h, 36.25% and 27.18% of MTX released at pH 7.4 and 5.3, respectively. Later on, MTX was released in a steady manner which was controlled by the diffusion process which is independent of polymeric matrices and concentration gradient [51]. In 13.5 h, 81.25% and 77.23% of MTX released in PBS at pH 7.4 and 5.3, correspondingly, which is in strong agreement with the US Pharmacopeia standard (USP XXIV) [52]. It is also evident from figure 12*b* that MTX was released in a more sustained manner at pH 5.3, which is favourable for *in vivo* delivery of MTX for cancer treatments. Moreover, MTX released was superior in slow release as reported in previous studies. For instance, Qindeel *et al.* fabricated poly(caprolactone)/poly(ethylene glycol) hydrogels that demonstrated 80% MTX released at pH 7.4 and 5 for rheumatoid arthritis [53]. Similarly, Zarbab *et al.* reported guar gum/chitosan/PVA hydrogels cross-linked with tetra orthosilicate hydrogels; 96% MTX was released in 7 h and 25 min in PBS at pH 7.4 for the treatment of colon cancer [54]. Nikitina *et al.* developed k-CG/MTX and k-CG/*β*-cyclodextrin hydrogels which showed 65% and 100% MTX released in 6 h in PBS at pH 7.4, respectively [55]. Fe_3_O_4_-incorporated k-CG/chitosan hydrogels were reported by Mahdavinia *et al*. The hydrogels reflected 68% of MTX released in 4 h in PBS at pH 7.4 and 5.3 [48]. Likewise, gelatin/PVA hydrogels were also explored for MTX released by Akhlaq *et al*.; in 12 h, 94.30%, 83.21% and 82.32% MTX released were detected in PBS at pH 1.2, 6.8, and 7.4 respectively [56].

### 3.13. Methotrexate release kinetics

**Figure 13.**
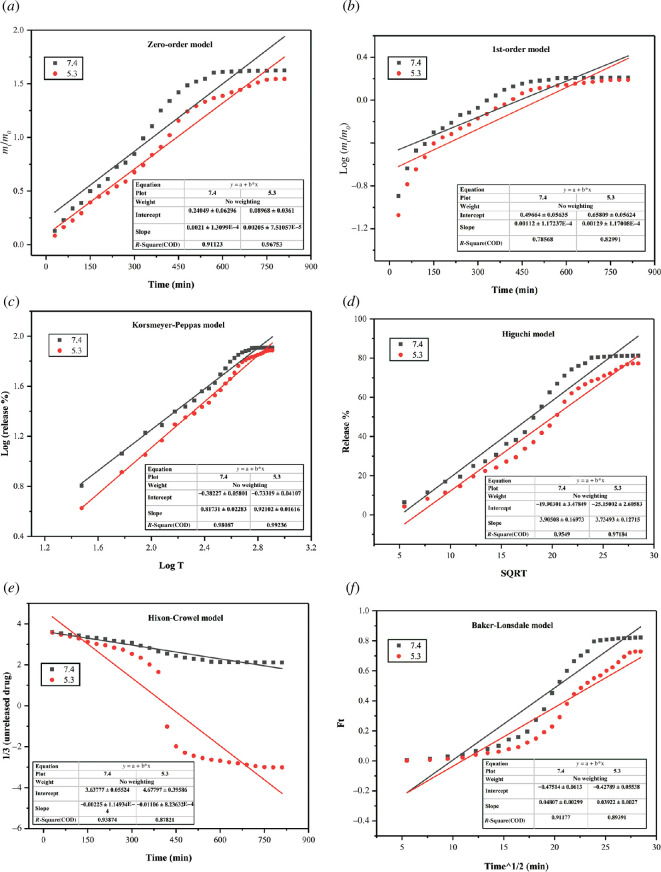
The outcome of different kinetic models for MTX release from MCAP-8.

In figure 13, the numerical data of drug release were fitted into kinetic models to confirm the transport mechanism of MTX from MCAP-8 by using equations (2.5–2.10). The values of *R* ^2^ in table 3 are greater than 0.85, which suggests that MTX released data best fitted to all models except first order. The values of slope from zero order, first order, Korsmeyer–Peppas, Higuchi, Hixon–Crowel and Baker–Lonsdale models at pH 5.3 are 0.02, 0.001, 0.921, 3.734, −0.011 and 0.0392, respectively. The value of *K*_o_ for zero-order reaction certified controlled MTX release. As in the present work, hydrogels are prepared by varying the amounts of APDMS and the value of *R*-square is greater than 90 for zero-order reaction rate, which suggests that MTX release from the APDMS cross-linked CG/SA/PVA/PAA hydrogels is independent to the concentration of APDMS. This trend is in accordance with the previously reported studies for MTX release [57,58].

**Table 3.** The values of *R*-squares for different kinetic models.

**m**odel	pH 5.3	pH 7.4
zero order	0.967	0.911
first order	0.829	0.786
Korsmeyer–Peppas	0.992	0.98
Higuchi	0.971	0.954
Hixon–Crowel	0.878	0.938
Baker–Lonsdale	0.89	0.911

The value of slope (*n*) is 0.921 computed by the Korsmeyer–Peppas model, which is greater than 0.89, which confirmed super case II transport of MTX from the hydrogel matrix [59]. The value of *n* 0.921 indicated that there is stress and transition of states in polymeric networks owing to the swelling, which are accountable for MTX release in PBS as also reported in the literature [54,60].

## 4. Conclusions

APDMS cross-linked, biodegradable cytocompatible and pH-responsive CG/SA/PVA/PAA hydrogels were prepared by solution casting technique. The syntheses of the hydrogel platform were confirmed by FTIR, TGA, SEM and AFM techniques. CAP-8 exhibited the highest 2471% swelling in distilled water. The swelling actions of hydrogels were decreased by the increase in APDMS from CAP-8 to CAP-32, which obeyed anomalous non-Fickian diffusion process. Hydrogels displayed maximum swelling at pH 7. This distinctive behaviour was explored for *in vitro* MTX released in PBS. As a result, 81.25% and 77.23% of MTX were released in PBS at pH 7.4 and 5.3, respectively. Korsmeyer–Peppas and zero-order models were best fitted for MTX release from MCAP-8, which verified sustained MTX release. From MCAP-8, MTX is released via the super case II mechanism. The fabricated hydrogels could be an efficient model platform for an effective delivery of MTX in the fight against cancer. However, *in vivo* investigations of MTX release are the main limitation and future perspective for CG-based hydrogels reported in the present study.

## Data Availability

The complete datasets are uploaded in DRYAD repository [61].
